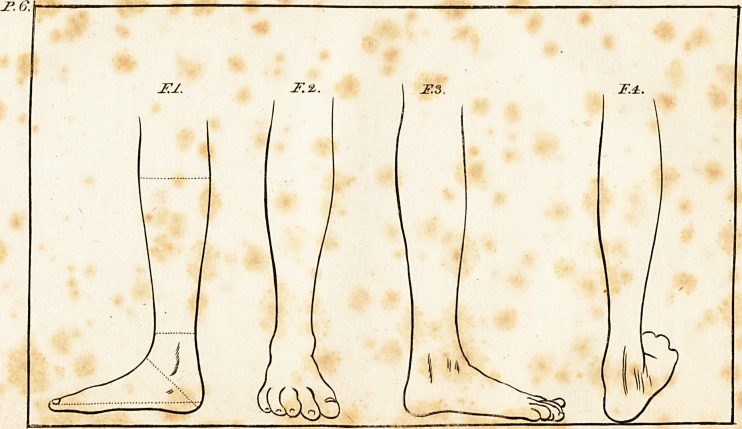# Mr. Sheldrake, on the Cure of Distortion

**Published:** 1801-01

**Authors:** T. Sheldrake

**Affiliations:** No. 50, Strand


### *To the Editors of the Medical and Phyſical Journal.*

GENTLEMEN,

IT has always appeared to me, that the publication of caſes, however important or authentic, ſhould be reſtricted to ſuch as tend to explain ſome remarkable fact, or determine ſome point of doctrine that is not perfectly ſettled. One of your correſpondents has lately advanced an opinion, that all diſtortions in the legs or feet, by whatever cauſe produced, *or how formidable soever they may appear*, may be perfectly cured by the application of one inſtrument. If this was ſo, it would materially ſhorten my labour in the communications I propoſe to ſubmit to you upon this ſubject, as I ſhould be authoriſed to conclude, that all diſtortions of the feet might certainly be cured by the very ſame means that were uſed to cure the patient whoſe caſe I related in my laſt; and by producing the ſpecific, put an end to all farther enquiry on the ſubject. But, as not one particle of the opinion, ſo confidently given, is true, I hope I ſhall be excuſed for ſending the hiſtory of another caſe of the ſame diſeaſe; by comparing which, with that you have already publiſhed, your readers will be competent to decide upon the truth and probability of that opinion.

There is another claſs of diſeaſes that is, in ſome reſpects, ſimilar to the above, viz. what are called contractions of the limbs, the conſequences of palſy, and other diſeaſes; as many of theſe may be cured by a ſkilful application of the ſame means that I have uſed in the cure of other diſtortions, I ſhall, in my next, relate a ſingular caſe of this deſcription, and then proceed to examine the theories that have been held, and the practice that has been founded on them, in attempting to cure theſe diſeaſes, and conclude with a full expoſition of my own.

I am, GENTLEMEN,

Your moſt obedient ſervant,

T. SHELDRAKE.

*No.* 50, *Strand,*

*December* 16, 1800.

P. S. Permit me to ſay, I ſhall ſend no anſwer to your correſpondent, Mr. Watt; for he has deſcended from diſcuſſing the ſubject in diſpute to *personal abuse, and is,* THEREFORE, *fallen too low for me to notice.*

### CASE II.

Sept. 7, 1798. A nephew of Mr. Duryer, No. 16, Dean Street, Holborn, was placed under my care for the cure of one diſtorted diſtorted foot; it happened before the birth; and from that period to the time I ſaw him, many unſucceſsful attempts were made to relieve him. He was, at this period, ſeven years old; the toes were of the natural form, but the whole metatarſus was diſtorted at its junction with the tarſus, and the foot was ſo twiſted from its natural poſition, that the toes pointed directly towards the oppoſite leg, and he ſtood upon a calloſity which had been formed on the outſide of the foot, upon the exterior cuneiform bone; the ſole of the foot was expoſed to view as he ſtood, and the whole foot was perfectly rigid, and the leg much waſted.

In four months the foot was ſo much altered, that it might be placed in its natural poſition while he ſtood upon it, but it was incapable of any voluntary motion; when he endeavoured to walk, it hung an uſeleſs appendage to the leg, and immediately fell into its original poſition.

In eight months more the foot was ſo far reduced to the natural ſtate, that he could walk upon it for a ſhort time in the natural way; but as it remained weak, and there was, after ſome time, a viſible tendency to relapſe, it was again bound up, and continued ſo for ten months more, when the bandages were laid aſide; and as, after ſix weeks trial, there appeared no tendency to relapſe, I finally took my leave of the patient.

It would be very eaſy to ſay, in few words, that this patient was completely cured; and thoſe, at leaſt, who ſhould ſee him walk flat upon this foot, and at the ſame time remember the ſtate it was in, would not diſpute the aſſertion; but as I think an accurate ſtatement of the facts will be more ſatisfactory, I truſt I ſhall be permitted to explain them.

I have one caſt taken from the foot in its originally diſeaſed ſtate; a ſecond, taken when it could be placed in the natural poſition, but remained flaccid and powerleſs, and therefore uſeleſs; a third, taken at the time he began to uſe the foot in the natural way; and a fourth, when I took my leave of him. By the aſſiſtance of theſe caſts, inſtead of referring to my own memory, or that of the child's friends, the one of which might be thought partial, and the other could not ſhow correctly what that diſeaſe was, which no longer exiſts, I am enabled to lay it before the reader in the moſt authentic and incontrovertible manner, by annexing correct drawings of the diſeaſe in its original, and the foot in its amended ſtate. The plate marked 3, contains four views of the diſeaſed foot; Fig. 1, is a front view of the leg, to ſhow how the foot was turned upwards and inwards with reſpect to it; Fig. 2, a back view of the leg for the ſame purpoſe; Fig. 3, a view of the outſide of the leg, in which no part of the metatarſus appears, becauſe it is turned directly directly inwards; and Fig. 4, is an inside view of the leg, to show the position of the toes in that point of view.

Whoever is acquainted with the structure and natural position of the bones of the foot, will perceive immediately upon inspecting these views, that there was no distortion of the bones of the metatarsus, individually, or with respect to each other, but that the metatarsus began to form an acute angle, turning directly inwards at its junction with the cuneiform bones: In these again, there appears to have been little distortion, either with regard to their mutual connection, or their connection with the astragalus; but the astragalus itself was so far removed from its natural situation, that instead of its circular head lying in the scaphoid cavity of the tibia, it lay in a direct perpendicular line with the fore part of the leg: the os calcis, instead of being placed upon the ground, lay in contact with the back part of the tibia; and the scaphoid cavity of this bone rested upon those parts of the tarsus and the astragalus, by which they were joined together: the result of this combination was, that the patient stood, and could only walk upon a callosity which was formed upon the outermost cuneiform bone. Add to this, that the distortion took place before the birth, and by remaining in this state till the patient was seven years old, the whole mass was so firmly connected, as to be perfectly rigid, and no part of it seemed endowed with any capacity for locomotion; an attention to these circumstances will be necessary, when we come to consider the treatment. Plate 4, contains four views of the leg, taken from the cast which was made after I quitted the patient. In making these views, I placed the leg in the same positions as in drawing the figures, with corresponding numbers in the former plate; of course, a comparison of the two plates will show exactly the difference between the foot in its diseased, and in its amended state.

I have only taken the extremes of this case, as the best method of showing the full effect of the alteration produced; it would be needless to give drawings of the intermediate casts of the foot, though it may be proper to observe, that in the second state of the case, when the foot could be placed in the natural position, but had no power to retain itself in it, it fell nearly into the form of the original disease, but the skin remained loose, and was universally wrinkled; in the third state, it approached nearer to the natural form, but still it had some distinguishing peculiarities; the instep was much higher, the shape of the ancles was not so well defined, and there was much loose skin about the ancle and outside of the foot; this afterwards disappeared; and when the last cast was taken, the foot measured five-eights of an inch more in length than it it did when the one before it was made. As this is more than can be accounted for from the natural growth of a natural formed foot in the ſpace of ten months, the ſurplus muſt be attributed to the alteration produced in the general ſtate of the foot during the progreſs of the cure.

I have hinted that this foot cannot be ſaid with propriety, to be *perfectly* reſtored to its natural ſtate. If Fig. 2 and 3, in Pl. 4, are examined, it will be ſeen that that part of the fibula which forms the outer ancle, is removed from its natural ſituation, and is quite cloſe to the tendo achillis; though this peculiarity does not render the foot unſerviceable, it certainly will never occaſion the leg to be admired for its beauty, and in this caſe I believe it to have been irremediable, though I have ſucceeded in removing that, as well as every other defect in younger patients. I believe it muſt have happened in the following way.

The fibula, from its length and poſition on the ſide of the aſtragalus, guards the joints ſo ſecurely from luxation outwards, that Mr. Pott ſaid, it was an invariable rule that this joint could not be ſo luxated without fracture of the fibula; perhaps he may be right: but it is certain that in this and ſimilar caſes, where the diſtortion takes place while the bones are in a cartilaginous ſtate, the head of the aſtragalus, by puſhing forwards, firſt drives the fibula outwards, and then forces it backwards towards the tendo achillis. To render the cure in this kind of caſe perfect, it ought to be undertaken while the bone is in a cartilaginous ſtate; in this ſtate I have ſeveral times cured it entirely: but in the caſe I have juſt related, I could not ſucceed in removing that particular defect, though it may not hereafter, in other caſes perhaps, prove irremediable.

## Figures and Tables

**F.1. F.2. F.3. F.4. f1:**
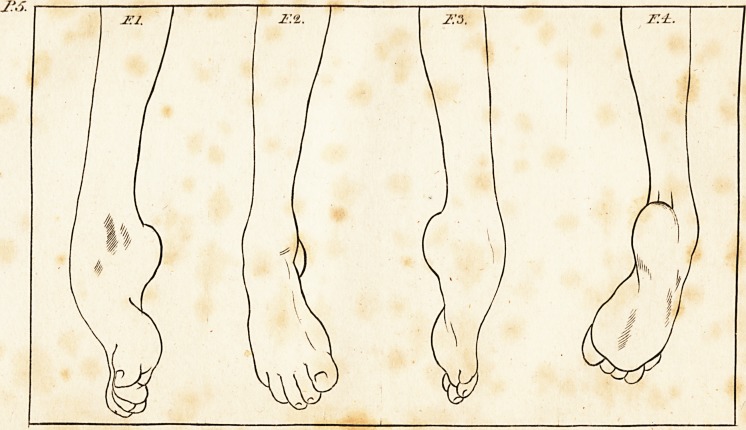


**F.1. F.2. F.3. F.4. f2:**